# Supplementary material for: Proliferation symmetry breaking in growing tissues
Source: bioRxiv. 2024 Sep 6:2024.09.03.610990. Preprint. [Version 1] doi: 10.1101/2024.09.03.610990 (PMC11398401; doi:10.1101/2024.09.03.610990)
Supplement: Supplement 1 [file media-1.pdf]

Supplemental Material

Proliferation symmetry breaking in growing tissues

Xinzhi Li, Aniruddha Datta, Shiladitya Banerjee

Department of Physics, Carnegie Mellon University, Pittsburgh, PA 15213, USA

I. VERTEX MODEL SIMULATIONS

Model Initialization and Parameterization. For the initialization of the vertex model simulation, we generated a set of random cell centers and constructed polygonal cells from voronoi tessellation. The initial state consisted of N_0 cells near the center of the system to provide a set of initial vertex positions under free boundary conditions. Each cell is assigned a preferred cell area A_0^i and polarity vector $\boldsymbol{p}_i$ in a random direction with motility speed v_0. Edges connected by vertices on the boundary cells act as the constraint from the external environment. The network topology is updated using T1 moves [1, 2] to allow neighbor exchanging and tissue expansion. Parameter values are summarized in Table I.

Cell growth and division. In Fig. S1a, we show snapshots of a growing tissue and how cell divisions are implemented in the simulation. Black dots represent cell centers and red dots indicate vertices obtained from voronoi tessellation. As shown in Fig. S1b, when a cell reaches the sizer/timer threshold, it is divided in the perpendicular direction of the main axis (indicated by the red line). The two dark red dots represent the two new vertices connecting the new edge separating the daughter cells which passes through the center of the mother cell. The preferred cell areas of daughter cells are calculated based on the fact that cell pressure remains the same before and after cell division. Assuming the mother cell has area A and preferred area A_0, the cellular pressure is $P = -K_A(A - A_0)$. Upon division, the daughter cells obtain area A_1 and A_2 where $A = A_1 + A_2$. Then the preferred areas of daughter cells are calculated as $A_0^1 = A_1 + P/K_A$ and $A_0^2 = A_2 + P/K_A$. This rule maintains pressure homeostasis within cells. G2 ages of the two daughter cells are reset to zero and the polarity vectors are randomly assigned. A representative single-cell area trajectory is shown in Fig. S1c.

The impact of tissue crowding on cell growth is evident in the single cell area tracks in Fig. S1c. During the early growth phase, cells displayed timer-like behavior, dividing almost at regular intervals of $\sim T$ because their size exceeded the G1 sizer threshold A_S. This resulted in size-reductive divisions until the cell size fell below A_S, at which point cells grew very slowly in the G1 phase and experienced complete cycle arrest due to their inability to transition to the timer phase. Such patterns of size reductive divisions have been observed experimentally [3]. The emergent patterns of proliferation can be controlled by changing cell elasticity K_A and contact inhibition parameter k, as shown in Fig. S2. At a fixed value of the contact inhibition parameter k, we observe more cells to be in G2 phase in the bulk by increasing K_A. In contrast, increasing k at a given K_A value, cells are more sensitive to crowding and fewer boundary cell divisions could occur. In the absence of contact inhibition, $k = 0$, tissue growth is homogeneous and divisions occur uniformly and isotropically (Fig. S3).

Cell polarity dynamics. In simulations of isotropic tissue growth, we suppress polarity alignment interactions. In that case, we model the dynamics of cell polarity vectors as undergoing rotational diffusion [4–6],

$$\begin{aligned} \partial_t \theta_i &= \eta_i(t), \\ \langle \eta_i(t)\eta_j(t')\rangle &= 2D_r\delta(t-t')\delta_{ij}, \end{aligned} \tag{1}$$

where θ_i is the polarity angle that defines $\boldsymbol{p}_i = (cos\ \theta_i, sin\ \theta_i)$, and $\eta_i(t)$ is a white noise process with zero mean and variance $2D_r$. The value of angular noise D_r determines the memory of stochastic noise in the system, giving rise to a persistence time scale $\tau = 1/D_r$ for the polarization vector $\boldsymbol{p}_i$. To induce anisotropic tissue growth, we implement polarity alignment dynamics that is discussed in the main text.

Characterization of tissue morphology. The irregular shape of the anisotropically growing tissue is measured by the ratio I_u/I_v, where I_u and I_v are the second moments of area along the major and the minor axes. For a polygon with n vertices $\{x_i, y_i\}$, numbered in counter-clockwise fashion, the second moment of area is given by [7, 8]

$$\begin{aligned} I_{xx} &= \frac{1}{12}\sum_{i=1}^{n}(x_i y_{i+1} - x_{i+1} y_i)(y_i^2 + y_i y_{i+1} + y_{i+1}^2) \\ I_{yy} &= \frac{1}{12}\sum_{i=1}^{n}(x_i y_{i+1} - x_{i+1} y_i)(x_i^2 + x_i x_{i+1} + x_{i+1}^2) \\ I_{xy} &= \frac{1}{24}\sum_{i=1}^{n}\{(x_i y_{i+1} - x_{i+1} y_i) \ldots. \\ & \quad (x_i y_{i+1} + 2x_i y_i + 2x_{i+1} y_{i+1} + x_{i+1} y_i)\} \end{aligned} \tag{2}$$

The centroidal moments are

$$\begin{aligned} I_{uu} &= I_{xx} - A y_c^2 \\ I_{vv} &= I_{yy} - A x_c^2 \\ I_{uv} &= I_{xy} - A x_c y_c \end{aligned} \tag{3}$$

The principle moments (I_1, I_2) and orientations can be obtained by solving eigenvalues and eigenvectors of the matrix

$$I = \begin{bmatrix} I_{uu} & -I_{uv} \\ -I_{uv} & I_{vv} \end{bmatrix}.$$

Then the shape of the polygon can be measured by the ratio of the principle area moments $A_R = I_1/I_2$. By analogy with single cells, we can also use the shape index of the tissue to measure the shape anisotropy,

which is defined as $q = P_t/\sqrt{A_t}$. Here P_t and A_t are the perimeter and area of the tissue, respectively. Lower ratio A_R and higher shape index correspond to more deformed irregular tissue shapes.

By calculating the moment of area and shape index of the tissue polygon formed by boundary vertices, we could characterize the irregularity of tissue shapes. As shown in Fig. S4a and c, at $\kappa_p = 0.05$, the ratio $A_R = I_1/I_2$ decreases while the shape index increases with the increasing of motility v_0, indicating tissue shapes become more anisotropic. At $v_0 = 0.1$, the ratio A_R (Fig. S4b) increases while the tissue shape index (Fig. S4d) decreases with the polarity alignment rate κ_p, representing more isotropic regular tissue shapes. When κ_p is increasing, the polarity vectors are more efficiently aligned, leading to collective drifting of the tissue which helps maintain regular isotropic tissue shapes. In the main text, we have discussed the indispensable role of contact inhibition in determining anisotropic growth patterns. Therefore, we show the phase diagram of $A_R = I_1/I_2$ and tissue shape index at various contact inhibition and cell motility values in Fig. S5. Active cell motility promotes anisotropic tissue shapes. In contrast, contact inhibition reinforces the uniformity of tissue growth and maintains regular shapes.

II. CONTINUUM MODEL

A. Model derivation and non-diemnsionalization

Here we describe the continuum model of tissue growth in one spatial dimension. This is relevant for planar tissue growth, with translational invariance along one of the in-plane axes. The tissue is characterized by three time varying fields, namely density $\rho(x,t)$, velocity $\mathbf{v}(x,t)$ and polarity $p(x,t)$. The density equation is derived by assuming mass conservation with logistic growth,

$$\partial_t \rho + \partial_x(\rho v) = \kappa\rho(1 - \rho/\rho_0) \tag{4}$$

where κ is the rate of proliferation and ρ_0 is the homeostatic density. Condition of force balance in the overdamped limit yields the following equation of motion,

$$\partial_x \sigma = \mu v - \mu v_0 p \tag{5}$$

where σ is the stress in the tissue, μ is the coefficient of friction, and v_0 is the speed of active cell motility. Active motility occurs along the direction of local cell polarity p. The constitutive equation for tissue stress is assumed to follow that of Maxwell viscoelastic materials since the tissue behaves as a fluid at long times due to cell rearrangements and divisions. We thus have,

$$(1 + \tau\partial_t)\sigma = \eta\partial_x v - \Pi(\rho) = \eta\partial_x v - (\Pi_0 + \chi^{-1}(\rho - \rho_0)) \tag{6}$$

where τ is the timescale of viscoelastic relaxation η is the viscosity and $\Pi(\rho)$ is the density-dependent pressure. We assume the pressure Π to be linear in density up to first order, with χ the compressibility. Putting the above two equations together gives the equation for cell velocity:

$$\mu(\tau\partial_t v + v) - \mu v_0(\tau\partial_t p + p) = \eta\partial_x^2 v - \chi^{-1}\partial_x\rho \; . \tag{7}$$

Polarity dynamics is governed by the following equation,

$$\partial_t p = \kappa_p \partial_x^2 p + [a(\rho - \rho_c) - bp^2]p \; , \tag{8}$$

where the first term in the RHS induces alignment of polarity, while the second term causes breaking of symmetry in the homogeneous steady state when the density is above the critical threshold ρ_c. The strength of alignment and the degree of symmetry-breaking are controlled by the parameters κ_p and a, respectively. We can non-dimensionalize Eqs. (4), (7) and (8) by defining rescaled variables as follows

$$t = \tau\bar{t}, \quad x = \sqrt{\frac{\eta}{\mu}}\bar{x} = \lambda\bar{x}, \quad \rho = \rho_0\bar{\rho}, \quad v = \frac{\lambda}{\tau}\bar{v}, \quad p = \frac{1}{\sqrt{\tau b}}\bar{p} \; . \tag{9}$$

These rescaled variables reduce the model equations to non-dimensional forms

$$\partial_{\bar{t}}\bar{\rho} = -\partial_{\bar{x}}(\bar{\rho}\bar{v}) + \bar{\kappa}\bar{\rho}(1-\bar{\rho}) \; , \tag{10.1}$$

$$\partial_{\bar{t}}\bar{v} = \partial_{\bar{x}}^2\bar{v} - \bar{v} - \bar{\chi}^{-1}\partial_{\bar{x}}\bar{\rho} + \bar{v}_0(\bar{p} + \partial_{\bar{t}}\bar{p}) \; , \tag{10.2}$$

$$\partial_{\bar{t}}\bar{p} = \bar{\kappa}_p\partial_{\bar{x}}^2\bar{p} + [\bar{a}(\bar{\rho} - \bar{\rho}_c) - \bar{p}^2]\bar{p} \; , \tag{10.3}$$

where

$$\bar{\kappa} = \kappa\tau, \quad \bar{\chi} = \frac{\eta\chi}{\rho_0\tau}, \quad \bar{v}_0 = \frac{v_0}{\lambda}\sqrt{\frac{\tau}{b}}, \quad \bar{\kappa}_p = \frac{\tau\kappa_p}{\lambda^2}, \quad \bar{a} = \rho_0\tau a, \quad \bar{\rho}_c = \frac{\rho_c}{\rho_0} \; . \tag{11}$$

B. Linear Stability Analysis

To determine the parameter regimes under which the local cell density remains bounded, we performed a linear stability analysis of the model equations about a homogeneous steady-state where polarity symmetry is broken. We linearized the fields as follows

$$\rho = \rho^*(t) + \delta\rho(x,t), \quad v = \delta v(x,t), \quad p = p^*(t) + \delta p(x,t) \; , \tag{12}$$

$$\partial_t\delta\rho = -\rho^*\partial_x\delta v + \bar{\kappa}(1-2\rho^*)\delta\rho \tag{13.1}$$

$$\partial_t\delta v = \partial_x^2\delta v - \delta v - \bar{\chi}^{-1}\partial_x\delta\rho + \bar{v}_0(\delta p + \partial_t\delta p) \tag{13.2}$$

$$\partial_t\delta p = \bar{\kappa}_p\partial_x^2\delta p + \bar{a}(\rho^* - \bar{\rho}_c - 3p^{*2})\delta p \tag{13.3}$$

We now perform a Fourier transform of the fields,

$$\mathcal{F}[\delta\rho] = \widetilde{\delta\rho} e^{i(qx-\omega t)}, \quad \mathcal{F}[\delta v] = \widetilde{\delta v} e^{i(qx-\omega t)}, \quad \mathcal{F}[\delta p] = \widetilde{\delta p} e^{i(qx-\omega t)} \tag{14}$$

which gives,

$$[\omega - i\bar{\kappa}(1-2\rho^*)]\widetilde{\delta\rho} - q\rho^*\widetilde{\delta v} = 0\,, \tag{15.1}$$

$$q\bar{\chi}^{-1}\widetilde{\delta\rho} - (\omega + i + iq^2)\widetilde{\delta v} + \bar{v}_0(\omega+i)\widetilde{\delta p} = 0\,, \tag{15.2}$$

$$[\omega + iq^2\bar{\kappa}_p - i\bar{a}(\rho^* - \bar{\rho}_c - 3p^{*2})]\widetilde{\delta p} = 0\,. \tag{15.3}$$

Solving for ω in terms of q from

$$\begin{vmatrix} \omega - i\bar{\kappa}(1-2\rho^*) & -q\rho^* & 0 \\ q\bar{\chi}^{-1} & -(\omega+i+iq^2) & \bar{v}_0(\omega+i) \\ 0 & 0 & \omega + iq^2\bar{\kappa}_p - i\bar{a}(\rho^* - \bar{\rho}_c - 3p^{*2}) \end{vmatrix} = 0 \tag{16}$$

gives

$$\omega = i[\bar{a}(\rho^* - \bar{\rho}_c - 3p^{*2}) - \bar{\kappa}_p q^2],\, -\frac{i}{2}[1+q^2-\bar{\kappa}(1-2\rho^*)] \pm \frac{1}{2}\sqrt{4\bar{\chi}^{-1}q^2\rho^* - [1+q^2-\bar{\kappa}(1-2\rho^*)]^2} \tag{17}$$

In order for the density to be bounded from above, we need $\mathrm{Im}(\omega) < 0$ which gives the following conditions

$$\bar{a}(\rho^* - \bar{\rho}_c - 3p^{*2}) - \bar{\kappa}_p q^2 < 0 \tag{18}$$

and

$$\bar{\chi} < \frac{4q^2\rho^*}{[q^2+1+\bar{\kappa}(1-2\rho^*)]^2}, \quad \bar{\kappa} < \frac{1+q^2}{1-2\rho^*} \tag{19.1}$$

Or

$$\frac{4q^2\rho^*}{[q^2+1+\bar{\kappa}(1-2\rho^*)]^2} < \bar{\chi} < \frac{q^2\rho^*}{\bar{\kappa}(1-2\rho^*)(1+q^2)}\,. \tag{19.2}$$

Setting $q = \pi/2L$, $\rho^* = 1$, $p^* = \pm\sqrt{a(\rho^* - \bar{\rho}_c)}$ the conditions become

$$a < \frac{1}{6}\left(1 - \sqrt{1 - \frac{3\bar{\kappa}_p\pi^2}{L^2(1-\bar{\rho}_c)}}\right) \quad \text{or} \quad a > \frac{1}{6}\left(1 + \sqrt{1 - \frac{3\bar{\kappa}_p\pi^2}{L^2(1-\bar{\rho}_c)}}\right) \tag{20}$$

$$\bar{\chi} > \frac{\pi^2}{L^2}\left[\bar{\kappa} - \left(\frac{\pi^2}{4L^2}+1\right)\right]^{-2} \tag{21}$$

where L is half the length of the domain of solution.

C. Numerical implementation and parameter choices

We solved Eqs. (10) numerically through the finite volume approach using the FiPy module of Python. We assumed a domain $[-L, L]$ of size $2L$, much larger than tissue size, with no-flux boundary conditions for all variables, ie

$$\partial_x \rho = \partial_x v = \partial_x p = 0 \quad \text{at} \quad x = \pm L \,. \tag{22}$$

The initial density was taken to be a narrow Gaussian distribution centered at the origin,

$$\rho(x, t = 0) = \frac{1}{2} \exp\left(\frac{x^2}{2\sigma^2}\right) \tag{23}$$

where the standard deviation σ (= 5 in our simulations) sets the initial size of the tissue. The initial polarity was randomly drawn from a normal distribution in the part of the domain within two standard deviations of the density profile, and zero elsewhere. The initial velocity was taken to be zero everywhere. The choice of model parameters for different simulation runs is summarized in Table II.

We determined the spatial extent of the tissue by stipulating that the density within the tissue is higher than a cutoff density of 0.01. Once we have determined the spatial extent of the tissue we can calculate its size, its midpoint and then the growth anisotropy index g_{ans}. During later stages of the simulation, the tissue starts collectively migrating in the direction of polarity and quickly reaches the boundary of the domain. To counter this behavior in the long simulation runs required for producing Fig. S8, we calculate the displacement of the tissue midpoint after every time step and displace all the variable profiles by an equal and opposite distance so the tissue remains centered at the origin. This does not affect the calculation of g_{ans}.

---

[1] Dapeng Bi, J. H. Lopez, J. M. Schwarz, and M. Lisa Manning. A density-independent rigidity transition in biological tissues. *Nature Physics*, 11(12):1074–1079, Dec 2015.

[2] Dapeng Bi, Jorge H. Lopez, J. M. Schwarz, and M. Lisa Manning. Energy barriers and cell migration in densely packed tissues. *Soft Matter*, 10:1885–1890, 2014.

[3] John Devany, Martin J. Falk, Liam J. Holt, Arvind Murugan, and Margaret L. Gardel. Epithelial tissue confinement inhibits cell growth and leads to volume-reducing divisions. *Developmental Cell*, 58(16):1462–1476.e8, 2023.

[4] Dapeng Bi, Xingbo Yang, M. Cristina Marchetti, and M. Lisa Manning. Motility-driven glass and jamming transitions in biological tissues. *Phys. Rev. X*, 6:021011, Apr 2016.

[5] Xinzhi Li, Amit Das, and Dapeng Bi. Mechanical heterogeneity in tissues promotes rigidity and controls cellular invasion. *Phys. Rev. Lett.*, 123:058101, Jul 2019.

[6] Thomas Fuhs, Franziska Wetzel, Anatol W. Fritsch, Xinzhi Li, Roland Stange, Steve Pawlizak, Tobias R. Kießling, Erik Morawetz, Steffen Grosser, Frank Sauer, Jürgen Lippoldt, Frederic Renner, Sabrina Friebe, Mareike Zink, Klaus Bendrat, Jürgen Braun, Maja H. Oktay, John Condeelis, Susanne Briest, Benjamin Wolf, Lars-Christian Horn, Michael Höckel, Bahriye Aktas, M. Cristina Marchetti, M. Lisa Manning, Axel Niendorf, Dapeng Bi, and Josef A. Käs. Rigid tumours contain soft cancer cells. *Nature Physics*, 18(12):1510–1519, Dec 2022.

[7] R. Soerjadi. On the computation of the moments of a polygon, with some applications. 1968.

[8] Carsten Steger. On the calculation of arbitrary moments of polygons. 1996.

Parameter	Symbol	Value						
		Fig. 1a-c	Fig. 2a-c	Fig. 2d	Fig. 2e	Fig. 3a-b	Fig. 3c	Fig. 3d
Initial cell number rate	N_0	1	9	9	9	9	9	9
Proliferation rate	G_0	0.5	0.5	0.5	0.5	0.5	0.5	0.5
Cell area elasticity	K_A	1	1	1-10	1	1	1-10	1
Contact inhibition	k	100	100	100	10-100	100	100	10-100
Variance of angular noise	D_r			1				
Edge tension	λ			-0.02				
Motility	v_0	0.1	0.2	0-0.3	0.1	0.1	0-0.3	0-0.3
Polarity alignment rate	κ_p	0	0.05	0.1	0.1	0.1	0.1	0.1
Sizer	A_S			2				
Timer	T			5				

TABLE I. Parameters for the vertex model simulations. The parameters are non-dimensional. In Fig. 3(a), the tissue without cell growth has $N_0 = 1001$ cells.

Parameter	Symbol	Value		
		Fig. 4a	Fig. 4b-c	Fig. 4d
Proliferation rate	$\bar{\kappa}$	0.5	0.5	0.5
Compressibility	$\bar{\chi}$	2	2	0.2 - 2
Motility	$\bar{v}_0$	5	5	1 - 5
Polarity alignment rate	$\bar{\kappa}_p$	0	1000	10
Symmetry breaking coefficient	$\bar{a}$	0	0.3	0.35
Threshold density	$\bar{\rho}_c$	0.2	0.2	0.2
Half-length of simulation box	L	100	100	50

TABLE II. Parameters for the continuum model. The first six parameters are nondimensional.

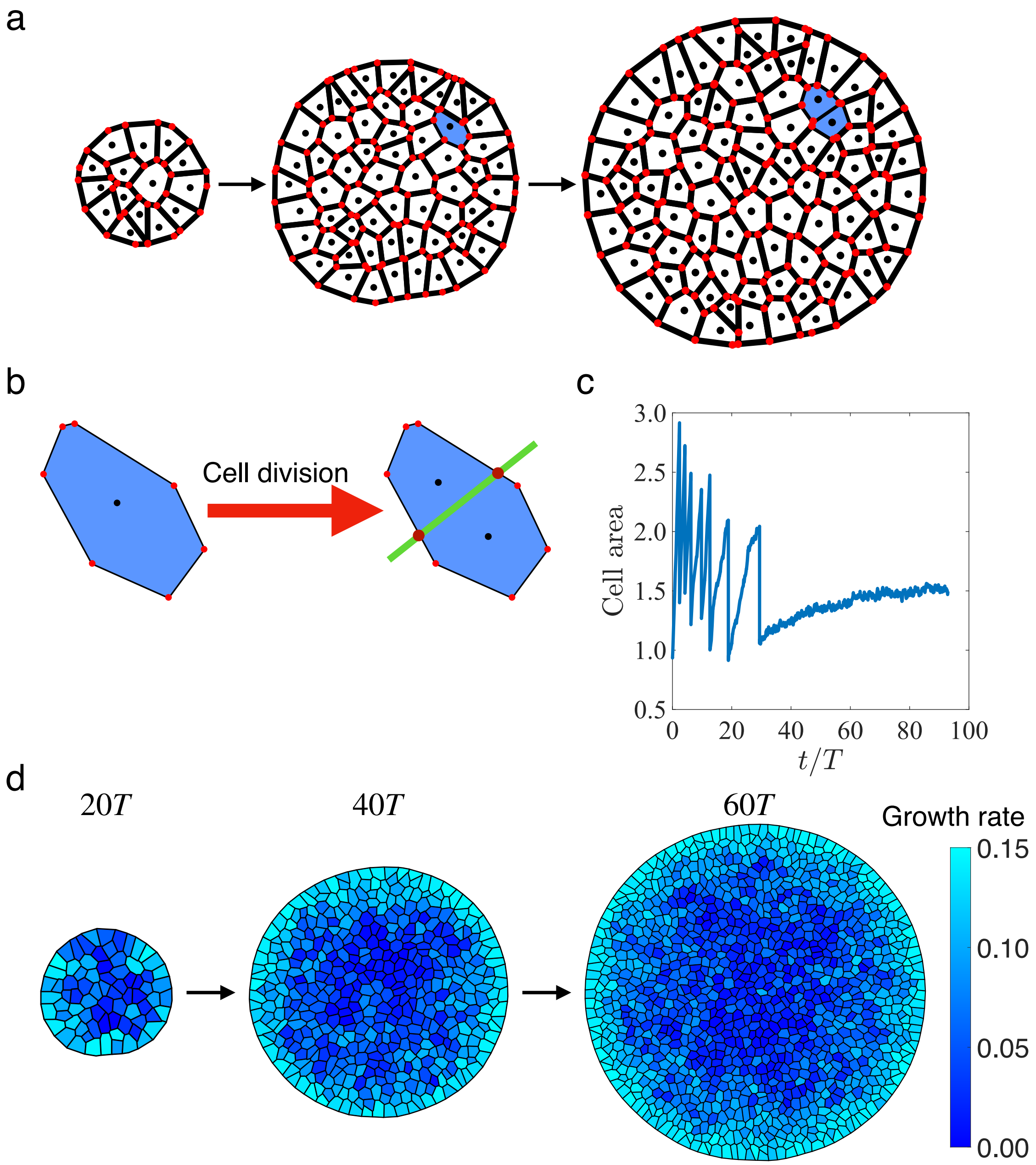

FIG. S1. **(a)** Time-lapse of configurations of an isotropically growing tissue. Black dots represent the cell centers and the red dots indicate the vertices obtained from voronoi tessellation. When the G1 sizer threshold is reached, a cell entering the G2 phase is marked by light blue in the snapshot. **(b)** Implementation of a cell division. When the cell reaches the timer threshold in the G2 phase, it divides in the direction perpendicular to the major axis (indicated by the green line). The two dark red dots represent the two new vertices connecting the new edge separating the daughter cells. **(c)** Single-cell area vs time for a representative cell in the bulk of the tissue, showing a transition to cell cycle arrest due to crowding-induced suppression of growth. **(d)** Timelapse of an isotropically growing tissue with random polarity. The colors represent cell growth rate which decreases from the boundary to the center of the tissue.

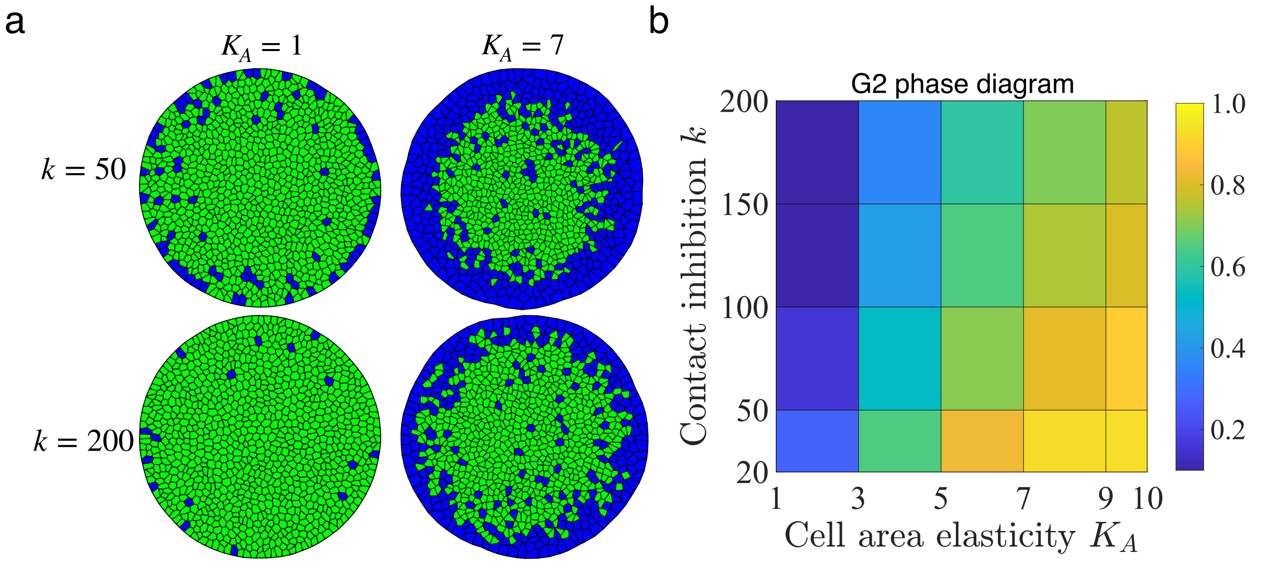

FIG. S2. **Patterns of cell cycle phase in a growing tissue by tuning contact inhibition k and cell area elasticity K_A.** **(a)** Snapshots of growing tissues exhibiting boundary and bulk growth. Green cells are in the G1 phase and cells in the G2 phase are colored in blue. **(b)** Phase diagram of the ratio of cells in G2 phase. At a fixed contact inhibition k, increasing K_A leads to more fraction of cells in the G2 phase. In contrast, increasing k at a given K_A value, cells are more sensitive to crowding and fewer cell divisions could occur in the tissue bulk, resulting in a lower fraction of cells in the G2 phase.

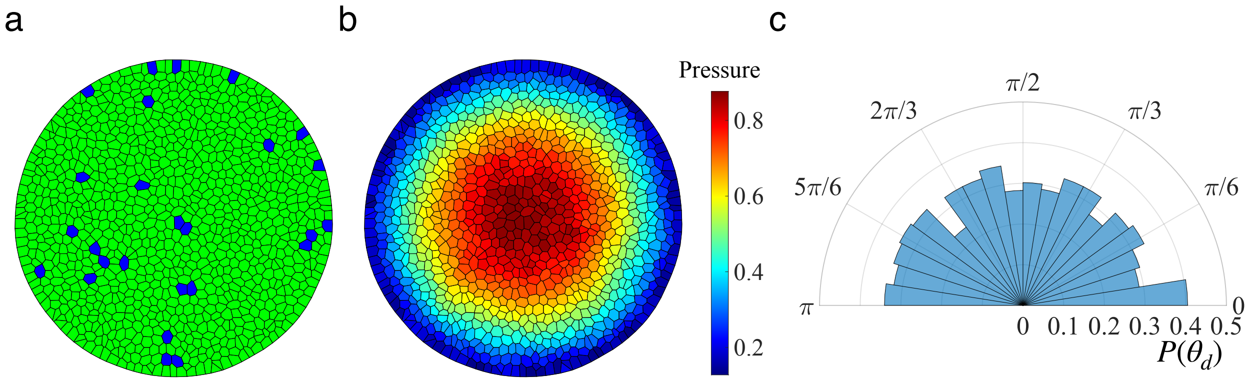

FIG. S3. **Growth pattern in the absence of contact inhibition.** Spatial map of **(a)** cells in G1 (green) and G2 (blue) phases and **(b)** pressure distribution for a growing tissue with $k = 0$. **(c)** The angular distribution of cell divisions shows isotropy.

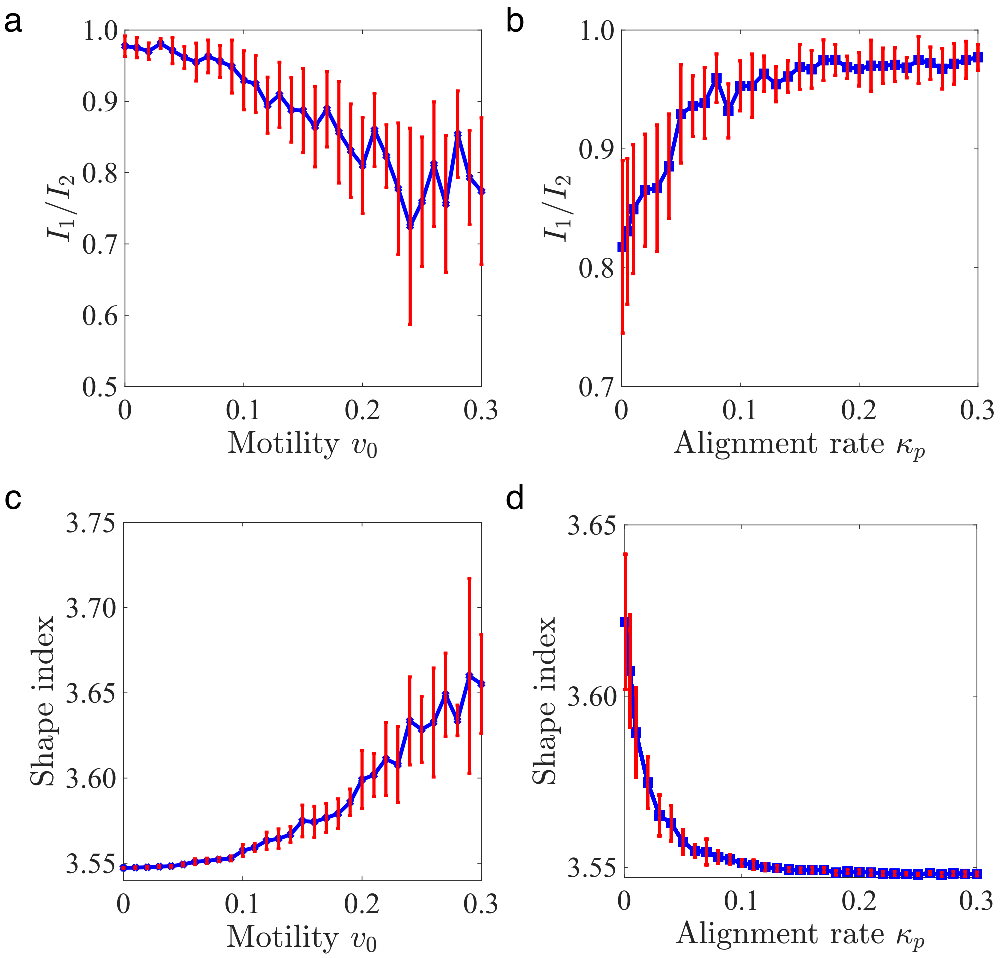

FIG. S4. **Control of tissue shapes by cell motility and polarity alignment rate.** At $\kappa_p = 0.05$, we characterize the tissue aspect ratio **(a)** and tissue shape index **(c)** as a function of cell motility v_0. With the increase of v_0, the cell division anisotropy increases and tissue shapes become non-round like. At $v_0 = 0.1$, the tissue aspect ratio **(b)** and tissue shape index in **(d)** is plotted as a function of the polarity alignment rate κ_p. When κ_p is increasing, the polarity vectors are more aligned, leading to the collective drifting of the tissue. The tissue shapes are more round-like.

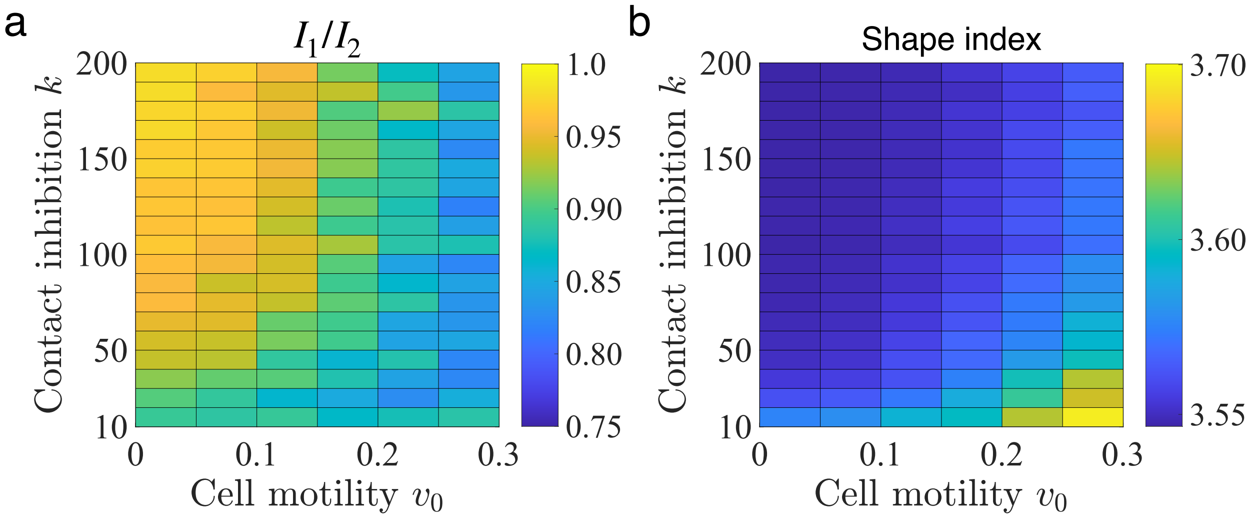

FIG. S5. **Characterization of tissue shapes at different values of cell motility and contact inhibition.** Phase diagram of tissue aspect ratio **(a)** and shape index **(b)** by tuning contact inhibition k and cell motility v_0. k suppresses while v_0 promotes the anisotropic tissue shapes.

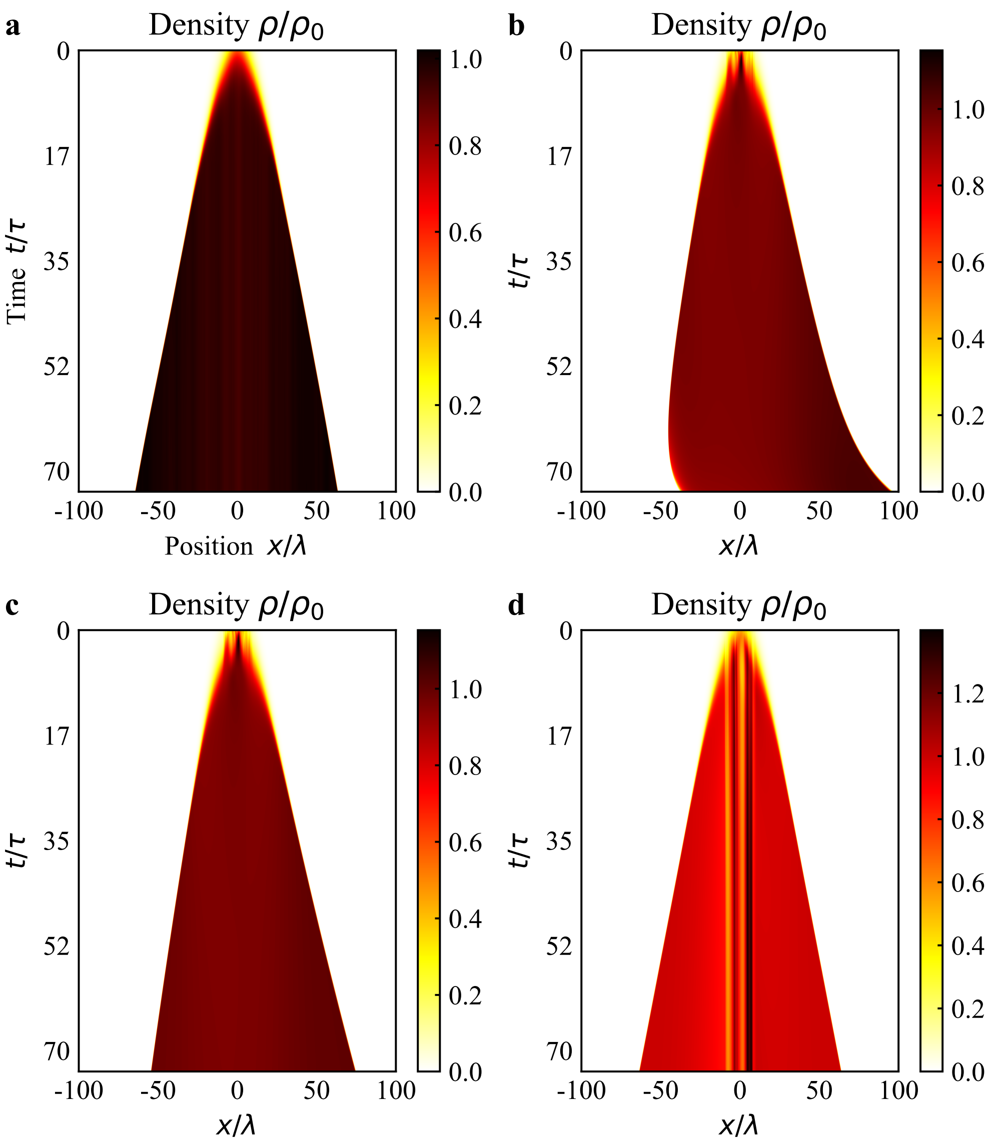

FIG. S6. **Effects of cell polarity on the pattern of growth.** When $\kappa_p = 0, a = 0$ **(a)** and polarity is randomised for every time step, growth is isotropic. When both κ_p and a are nonzero ($\kappa_p = 1000$, $a = 0.3$ **(b)**) the tissue grows anisotropically. For $\kappa_p = 1000$, $a = 0$ **(c)** the anisotropy is much less pronounced. In the presence of only the symmetry breaking term ($\kappa_p = 0$, $a = 0.3$ **(d)**) the initial fluctuations in the density, due to the initial random configuration of polarity, persist through time.

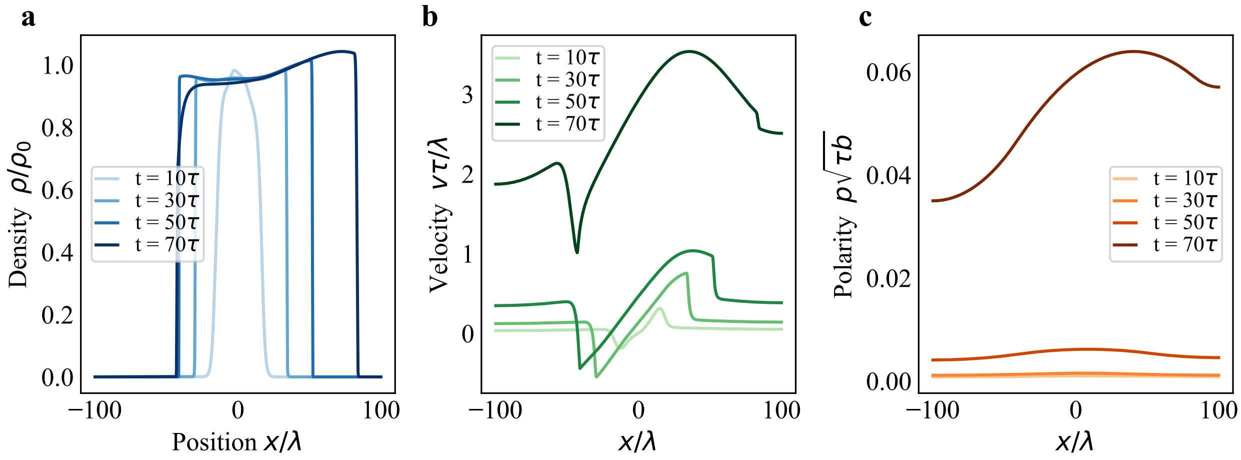

FIG. S7. **Spatial profiles of density, polarity and velocity during anisotropic growth.** Snapshots of **(a)** Density, **(b)** Velocity and **(c)** Polarity during different times for the anisotropically growing tissue shown in Fig. 4b and Fig. S6b.

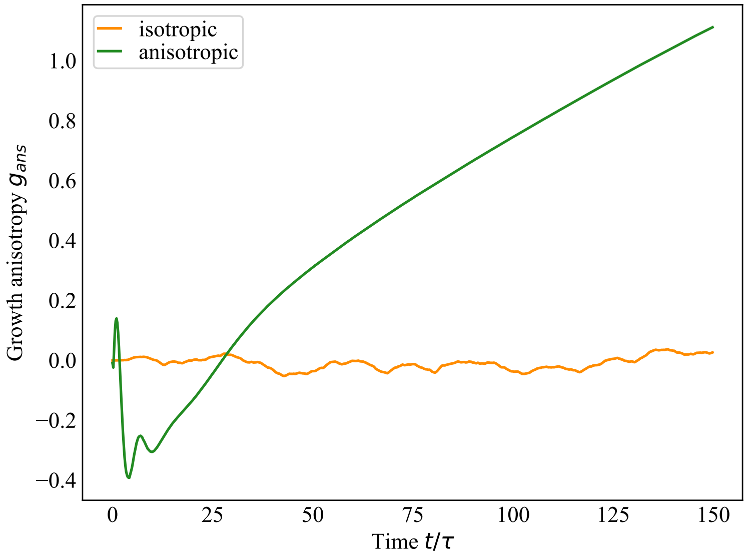

FIG. S8. **Growth anisotropy index for isotropic and anisotropic growth.** The growth anisotropy index $g_{ans} \approx 0$ throughout the simulation for isotropically growing tissue, but keeps increasing for the anisotropic case after some initial fluctuations.
